# Commentary: The effects of acupuncture on patients with premature ovarian insufficiency and polycystic ovary syndrome: an umbrella review of systematic reviews and meta-analyses

**DOI:** 10.3389/fmed.2025.1551033

**Published:** 2025-09-24

**Authors:** Haomin Sun, Jianqiao Fang, Xiaomei Shao

**Affiliations:** Key Laboratory of Acupuncture and Neurology of Zhejiang Province, The Third Affiliated Hospital of Zhejiang Chinese Medical University, The Third Clinical Medical College, Zhejiang Chinese Medical University, Hangzhou, China

**Keywords:** commentary, acupuncture, premature ovarian insufficiency, polycystic ovary syndrome, systematic reviews and meta-analyses, critical interpretive synthesis

## Introduction

We read with interest the study by Tianyu Bai et al. entitled “The effects of acupuncture on patients with premature ovarian insufficiency and polycystic ovary syndrome: an umbrella review of systematic reviews and meta-analyses” (1). Indeed, because of the large fluctuations in follicle stimulating hormone (FSH), the diagnosis and prognosis of premature ovarian insufficiency (POI) lacks a stable indicators (2, 3). In addition, In 2017, JAMA published a Chinese randomized clinical trial (RCT) suggesting that acupuncture does not improve live birth rates in Chinese patients with polycystic ovary syndrome (PCOS) and does not support acupuncture for infertility in such patients (4). In this meta-analysis, Tianyu Bai et al. reported that in patients with PCOS, acupuncture therapy was significantly associated with higher pregnancy rates, ovulation rates, and lower serum luteinizing hormone (LH), testosterone, LH/follicle stimulating hormone (FSH), insulin resistance, and BMI levels. For POI, acupuncture significantly improved serum FSH, LH, LH/FSH ratio, and estradiol levels. They also proposed that acupuncture may improve hormone imbalance in patients with POI through improvement of ovarian function, modulation of the hypothalamic–pituitary-ovarian (HPO) Axis, and reduction of stress by two basic research studies on PCOS. The original authors therefore concluded that acupuncture-related therapies may improve pregnancy rates and metabolic and hormonal imbalances in patients with POI and PCOS (1).

## Discussion

Firstly, the pathophysiology of POI and PCOS is different, which may increase the heterogeneity between studies, complicate the analysis process, and reduce the reliability and accuracy of the results (5, 6). The final results of the article are positive, but the mechanistic pathways behind these results, in particular the differences between acupuncture treatment in POI (marked by follicular depletion and hypoestrogenism) and PCOS (characterized by follicular excess and hyperandrogenism), have not been fully explored. A more in-depth analysis of these perspectives may shed light on why similar interventions produce different outcomes, thereby guiding the development of treatments for these clinically distinct disorders.

In addition, the authors mention that recent meta-analyses on the therapeutic effects of acupuncture in women with POI and PCOS have provided conflicting results with positive (7) or ineffective (8) results. Also, in their final conclusion, the authors mention the need for large-scale RCTs with rigorous methodological standards so that the role of acupuncture in these patients can be better understood through systematic reviews and meta-analyses. Therefore, in the context of the current lack of high-quality RCTs and the different meta-analyses showing different results, applying critical interpretive synthesis (CIS) to provide clinicians and patients with more comprehensive guidance may be a more appropriate approach (9, 10). CIS not only focuses on the aggregation of data, but also provides a deeper understanding of the complexity of the findings through critical analysis (11). At the same time, CIS can also provide theoretical directions and research ideas for further studies, guiding researchers to explore new areas of acupuncture treatment in depth (12). Authors can also combine the two methods of systematic review and meta-analysis and CIS (13) to complement each other's strengths and weaknesses, so as to assess the efficacy and application value of acupuncture in these two diseases in a more comprehensive and in-depth manner.

The authors performed searches of many databases, which is commendable. However, the authors' search terms were not comprehensive enough compared to previous acupuncture studies (14, 15). The search terms for acupuncture as a primary intervention should also include the words such as acupoints, meridian, and warm needling, and perhaps these would have also searched for some higher quality studies for this systematic evaluation and meta-analysis. In addition, the Chinese databases could also be searched for TCM terms for diseases in order to search more carefully for all the studies needed (16, 17).

We applaud the strengths of this umbrella review of systematic reviews and meta-analyses, including the large number of studies analyzed, the large sample size, and the examination of a variety of clinically-important outcomes. In addition, although the authors also performed subgroup analyses based on the type of treatment, given that individual patient characteristics, such as BMI, disease severity, and genetic factors, may influence the response to and outcome of acupuncture treatment (18, 19). It would be valuable to analyze how these individual factors interact with acupuncture and influence treatment outcomes in future studies (18, 20). This could involve subgroup analyses based on these characteristics to identify subgroups of patients more likely to benefit from acupuncture.
